# Robotic percutaneous coronary intervention (R-PCI): Time to focus on the pros and cons

**DOI:** 10.1016/j.ihj.2023.04.002

**Published:** 2023-04-18

**Authors:** E. Thirumurugan, K. Gomathi, P. Swathy, Syed Ali Afrin, R. Karthick

**Affiliations:** a Srinivas University, India; b College of Allied Health Science, DR MGR Educational and Research Institute, ACS Medical College, Chennai, Tamil Nadu, India

**Keywords:** Robotics, Coronary artery disease, Radiation exposure, Telestenting, Remote robotic technology

## Abstract

**Aim:**

To assess the safety, efficiency, and device compatibility of the Second Generation Robotic System.

**Methods:**

Data on Robot-Assisted PCI (R–PCI) is frequently insufficient in India. Many articles were published in national, non-indexed journals that are not available online and are difficult to obtain. Recognizing these constraints, the current review is intended to compile the available data on this important new innovation technique. This review could encourage future research and serve as a valuable source of information.

**Results/Conclusion:**

In terms of procedure efficiency, operator radiation reduction, and safety, the recent implementation and development of second-generation robotic systems have had a significant impact on interventional cardiology. This technology will play a significant role in the future of interventional cardiology as advancements eliminate the need for manual assistance, improve devices compatibility, and expand the use of robotics for telestenting procedures. A larger study demonstrating the safety and feasibility of tele-stenting over greater geographic distances, as well as addressing fundamental technical difficulties, would be required before attempting R–PCI.

## 1 Introduction

In 1977, Gruntzig and colleagues performed the first percutaneous coronary intervention (PCI).^1^ Recent developments in PCI technique, such as atherectomy devices, periprocedural pharmacotherapy, drug-eluting stent development, and intracoronary imaging, have led to a dramatic increase in the use of PCI for coronary revascularization in cardiology.^2^ Despite this innovation, the essential components of the PCI technique remain unchanged. The radiation exposure that PCI operators are subjected to can cause cataracts and cancer because it is so harmful. Robotic technologies have been developed to alleviate the burden of radiation exposure by relieving operators from the tableside.^3^ This state-of-the-art review outlines the safety, efficiency, and device compatibility of the Second Generation Robotic System.

## 2 Rationality of the review

Data on robotic PCI (R–PCI) in India is often insufficient. Some articles were published in journals that are not indexed in databases and are not easily found online. The review focuses on this new innovation technique, which is still in its early stages. So far, there is limited information about it. This review could encourage future research and serve as a valuable source of information.

## 3 CorPath 200 ROBOTIC PLATFORM (Corindus Inc., a Siemens Healthineers Company, Waltham, USA)

### 3.1 Procedure related (first generation)

A fundamental R–PCI procedure is carried out similarly to a manual process. After obtaining vascular access, an interventional cardiologist inserted a 6-French introducer sheath via the radial or femoral arteries. The in-lab experts loaded a sterile guide catheter, guidewire, and balloon catheter into the robotic cassette. The primary operator may depart from the table and sit in the CorPath control cockpit. Pushing on the joystick control advances the guidewire through the guide catheter and into the coronary artery. Once the wire is in place, the catheter can be advanced with the previously loaded balloon. An operator at a table can use extension tubing and lead shielding to inflate and deflate a balloon to lower his or her radiation exposure risk. The same techniques are used to connect, reposition, and deploy a coronary stent.

**PROS:** Removing the operators from the tableside can help to decrease radiation exposure.

**CONS:** The absence of active guide catheter control is a severe drawback of the CorPath 200 system.^4^

## 4 CorPath GRX ROBOTIC PLATFORM (Corindus Inc., a Siemens Healthineers Company, Waltham, USA)

### 4.1 Procedure related (second generation)

The CorPath GRX system uses a separate drive inside the robotic cassette to facilitate active guide catheter control, the FDA-approved next-generation robotic platform. After the initial manual engagement, the operator actively advances or retracts the guide catheter and conduct rotations in both the clockwise and counterclockwise directions.

**PROS:** The GRX system's touchscreen monitor is another feature that makes it more user-friendly by providing step-by-step instructions for removing and exchanging equipment. The operator is at the table-side less often to adjust the guide catheter.

**CONS:** Due to the unusual nature of the treatment, the in-lab interventional cardiologist and a cath lab technologist were on standby to provide emergency support.^5^

## 5 Procedural safety

Rafael Beyar et al (2006) recently conducted a study on 18 patients with confirmed CAD treated by PCI. The author defined study endpoints as successfully navigating the guidewire across the lesion and precisely positioning the device. The author defined technical success as completing the procedure without resorting to manual mode. The same author defined clinical success as completing the procedure without complications (30% residual stenosis or without in-hospital MACE).^6^ Granada et al (2011) defined major adverse cardiac events (MACE) as cardiac mortality, myocardial infarction with or without Q waves, or clinically induced target vessel revascularization.^7^ Byomesh Tripathi r et al (2021) defined separate endpoints for safety, including contrast use, fluoroscopy time, and radiation exposure.^8^ (Table 1, Table 2).

**Table 1:** Safety of first-generation robotic platform in performance.

CorPath 200 ROBOTIC PLATFORM (First Generation)					
**Procedural Safety:**		Granada et al (2011).^7^	Weisz et al, (2013).^9^	Mahmud et al (2017).^10^	J. Harrison et al,(2017).^11^
1.Study end points		100%	–	–	–
2. Technical success		97.9%.	98.8%	91.7%	81.5%
3. Clinical success		100%.	97.6%	99.1%	99.1%
4. Separate-end points for safety	**Contrast use**	158.8 ± 53.8	144.2 ± 70.4	183.4 ± 78.8	170.0 ± 63.6
	**Fluoroscopy time**	11.5 ± 3.7	11.1 ± 6.2	18.2 ± 10.4	15.2 ± 7.2
	**Radiation exposure. (reduction)**	97.1%	95.2%	**-**	–

**Table 2:** Safety of second-generation robotic platform in performance.

CorPath 200 ROBOTIC PLATFORM (Second Generation)						
**Procedural Safety:**		Smitson et al (2018).^12^ (N-40)	DOU KF, et al (2019).^13^ (N-10)	Yoshiaki Mitsutake et al(2021).^14^ (N-28)	Lemos et al (2022).^15^ (N-83)	Seifert M et al, (2022).^16^ (N-71)
1.Study end points		–	–	–	–	–
2. Technical success		90.0%.	100%	90.0%	85.7%	94.2%
3. Clinical success		97.5%.	100%	93.3%	–	–
4. Separate-end points for safety	**Contrast use**	171.6 ± 64.6	127.0 ± 40.3	93.2 ± 44.5	206.4 ± 114.4	145
	**Fluoroscopy time**	17.4 ± 5.8	18.2 ± 8.0	27.5 ± 18.9	–	20.4
	**Radiation exposure. (DAP,Gy Cm**^**2**^**)**	7162 ± 5424	**-**	**-**	**-**	2298

## 6 Supporting robotic-assisted percutaneous coronary intervention: the evidence

### 6.1 First generation

Several prospective studies and clinical trials have examined the robotic platform **(**Table 1, Table 2**)**. In 2006, a study showed that the CorPath 200 was safe to use during coronary procedures. Beyar and colleagues were the first to demonstrate robot-assisted PCI in human use. The operator performed the procedure safely and precisely from a distance. In a new remote navigation system pilot study, 18 patients with uncomplicated coronary lesions were treated with robot-assisted PCI. It showed 100% clinical success, 94% guidewire success, and 83% total success in robot-assisted operations.^6^

In the PRECISE study, Weisz and colleagues evaluated the safety and clinical effectiveness of the CorPath 200 robotic system. In this study, 164 subjects had R–PCI on predominantly superficial coronary lesions (87.2% were type A). The clinical success rate in the PRECISE study was 97.6%. In comparison, the technical success rate was 98.8%, indicating that R–PCI was a safe and effective intervention method in primarily superficial lesions.^9^ Poor support of guidewires and guide catheters was a significant cause of the failure of the first-generation robotic system and the subsequent need for manual support during the procedure. Other considerations for conversion included the use of interventional techniques that were beyond the abilities of the robotic platform, as well as, in rare cases, adverse consequences.

### 6.2 Second generation

The CorPath GRX allows operator manipulation from the robotic cockpit, which aids in resolving the guide catheter support issue. Due to this, the Smitson and colleagues study was created. It was a prospective, open-label, single-centre safety and feasibility study of R–PCI that assessed the efficiency and safety of the CorPath GRX System. In this study, 40 subjects had R–PCI on 77.8% (American College of Cardiology/American Heart Association) type B2/C coronary lesions. The clinical success rate in this study was 97.5%, and the technical success rate was 90.0%, proving that R–PCI was a secure and reliable intervention method for most lesions.^12^

#### 6.2.1 ACC/AHA lesion classification

Coronary artery plaque burden is classified using the ACC/AHA classification of coronary lesions.^17^ Robotic-assisted PCI is safe and effective for treating a wide range of coronary artery disease cases, from mild to severe. The technical and clinical success rate is over 90%, as demonstrated in Table 3.

**Table 3:** R–PCI performance in various types of lesion severity according to ACC/AHA lesion classification.

Lesion classification	Smitson et al (2018).^12^ (N-40) (L = 54)	DOU KF, et al (2019).^13^ (N = 10) (L = 11)	Yoshiaki Mitsutake et al(2021).^14^ (N-28) (L = 48)	Lemos et al (2022).^15^ (N-83) (L = 112)	Seifert M et al, (2022).^16^ (N-71) (L = 86)
A	1.8%	–	–	–	1.2%
B2/C	–	–	47.9%	–	–
B1	20.4%	36%	–	22.3%	10.5%
B2	16.7%	27%	–	18.8%	18.6%
C	61.1%	36%	–	58.9%	69.8%
Technical and Clinical success	>90%	>90%	>90%	>85%	>90%

#### 6.2.2 Equipment feasibility

Although coronary robotic systems allow for balloon manipulation, two critical steps of the procedures—gaining arterial access and manipulating the guiding catheters—are still done manually. The robotically assisted system works with all 5- to 7-F guiding catheters, 0.014-inch guidewires, rapid-exchange balloon and stent delivery systems, and all standard catheterization lab supplies and imaging equipment (Table 4). Robot-assisted PCI suites frequently use accessories like extension tubing and power injectors for contrast delivery. Fluoroscopic, electrocardiographic, and hemodynamic images are duplicated on monitors inside the cockpit, allowing the operator to view them from a close distance and at eye level.

**Table 4:** Robotic-Assisted Percutaneous Coronary Intervention equipment feasibility.

Procedural Safety:	Smitson,et al (2018).^12^ (N-40)	DOU KF, et al (2019).^13^ (N-10)	Yoshiaki Mitsutake et al (2021).^14^ (N-28)	Lemos et al (2022).^15^ (N-83)	Seifert M et al, (2022).^16^ (N-71)
1.Introducer needle (Gaining-arterial access)	∗	∗	∗	∗	∗
2. Sheath introducer	∗	∗	∗	∗	∗
3.Guide catheters	∗	–	∗	∗	∗
4.Guidewire	∗∗	∗∗	∗∗	∗∗	∗∗
5.Balloon catheter	∗∗	∗∗	∗∗	∗∗	∗∗
6.Stents	∗∗	∗∗	∗∗	∗∗	∗∗
7.Radioopaque dye delivery	–	–	∗∗	–	∗

(Note- ∗ - Manual and ∗∗ - Robot-assisted PCI).

##### 6.2.2.1 Imaging modality: IVUS

In order to demonstrate the effectiveness of intravascular imaging-guided R–PCI without any challenges, Yorihiko Koeda et al performed 110 R-PCIs using the CorPath GRX from 2019 to 2021 in Japan. In this study, intravascular imaging-guided R–PCI resulted in favourable initial treatment outcomes and was carried out without complications. However, the CorPath GRX cannot control all types of intravascular imaging catheters, which were manually controlled in this study.^18^ More recently, Arif A. Khokhar and colleagues evaluated the key technological advancements in the most recent robotic CorPath GRX system and reported that IVUS was carried out robotically using the Eagle Eye Platinum catheter (Philips) in a complex left main to the left anterior descending (LAD) artery lesion.^19^ The robotic system is not capable of manipulating rotational IVUS systems but can do phased array IVUS; however, phased array generally operates at a lower frequency and therefore has lower resolution.

##### 6.2.2.2 Other devices

The inability of CorPath GRX to manage specific devices, such as fractional flow reserve (FFR) and thrombus aspiration, rotational atherectomy devices, embolic protection devices, and intravascular imaging equipment (OCT), resulted in conversion to manual intervention. The main objective of robotic technology for PCI has been to lessen workplace risks for operating physicians. The emphasis must shift to the patient's advantages. This will necessitate the ability to include additional interventional devices.

##### 6.2.2.3 Laser atherectomy

Almasoud and colleagues used the ELCA catheter's RX function to perform the first demonstration of a robot-assisted laser atherectomy. The study found that laser atherectomy is an effective adjunctive lesion modification technique that can precisely control the catheter used during atherectomy.^20^

##### 6.2.2.4 Diagnostic catheter

Swaminathan and colleagues performed the first-in-human demonstration of a robot-assisted coronary angiogram using a diagnostic catheter. According to the findings of this study, the Corpath GRX robotic system (Corindus, Waltham, MA) used a 6F diagnostic catheter to provide compatibility with adequate support for catheter manipulation. Catheters with a smaller diameter would be more prone to inadequate tracking and weaken with repeated rotations.^21^ Recently, Ryan D. Madder and colleagues evaluated the efficacy and safety of robotic diagnostic coronary angiography and found that 100% of procedures were successful and had no significant complications.^22^

##### 6.2.2.5 Microcatheters

Hirai and colleagues performed the first-in-human demonstration of a robot-assisted system on CTO patients. This study found that the Corpath GRX (Corindus, Waltham, MA) robotic system is only compatible with quick exchange devices. The hybrid algorithm crossed the CTO segment manually using microcatheters and over-the-wire CTO-PCI devices. In this trial, however, the primary operator radiation was reduced by 48% compared to manual CTO PCI.^23^

### 6.3 Use of robotic system during pandemic as an alternative

Lemos and colleagues made the first-in-human demonstration of a robot-assisted system in COVID-19 patients. To minimize patient proximity to the medical staff, robotic-assisted PCI was carried out by unscrubbed cardiac interventionalists from a control cockpit outside the catheterization suite. In this study, R–PCI resulted in favourable initial outcomes and was carried out without complications.^15^

### 6.4 Tele communication

T.M. Patel and colleagues performed the first-in-human demonstration of a telerobotic long-distance R–PCI intervention using balloon angioplasty and stent deployment interventions from a remote location. According to the study, remote tele-R-PCI using the CorPath GRX telerobotic platform is possible with successful results if nearby cardiac catheterization facilities and reliable network access are both available.^24^ Ryan D. Madder and colleagues used the PCI simulator and a robotic drive to remotely manipulate coronary devices over more than 3000 miles (transcontinental) and 206 miles (regional). The clinical success rate in this study was 100% in both regional and transcontinental models. According to the study results, the incredible distance characteristic of the transcontinental model with no relation to procedure time significantly differs from the regional model for cases performed on wired or non-wired 5G, demonstrating that remote stenting by R–PCI is a safe and reliable method of intervention for superficial lesions.^25^

### 6.5 Presumed reduction in radiation exposure and orthopaedic injuries by using R–PCI

PCI complexity has steadily increased with other technological advances, leading to interventional cardiologists spending more extended periods in lead aprons, significantly impacting the musculoskeletal system. The Society for Cardiovascular Angiography and Interventions (SCAI) has demonstrated nearly 50% incidence of occupational-induced orthopaedic injuries throughout an operator's career.^26^ Interventional cardiologists have two-to three-times higher radiation exposure per year than radiologists, with a typical cumulative lifetime attributable risk of one cancer (both fatal and nonfatal) per 100 exposed subjects.^27^ Robotic-assisted PCI offers a safer way for interventionists to protect themselves from radiation exposure by removing the primary operator from the table-side during the procedure. Several studies have shown that R–PCI reduces operator radiation exposure.^9^^,^.^12^^,^.^10^^,^.^11^ In addition to greatly reducing operator radiation exposure, robotic interventions allow the operator to eliminate leaded aprons and sit down during the procedure, reducing orthopaedic injury and operator fatigue.

### 6.6 Future directions

**Fig. 1:**
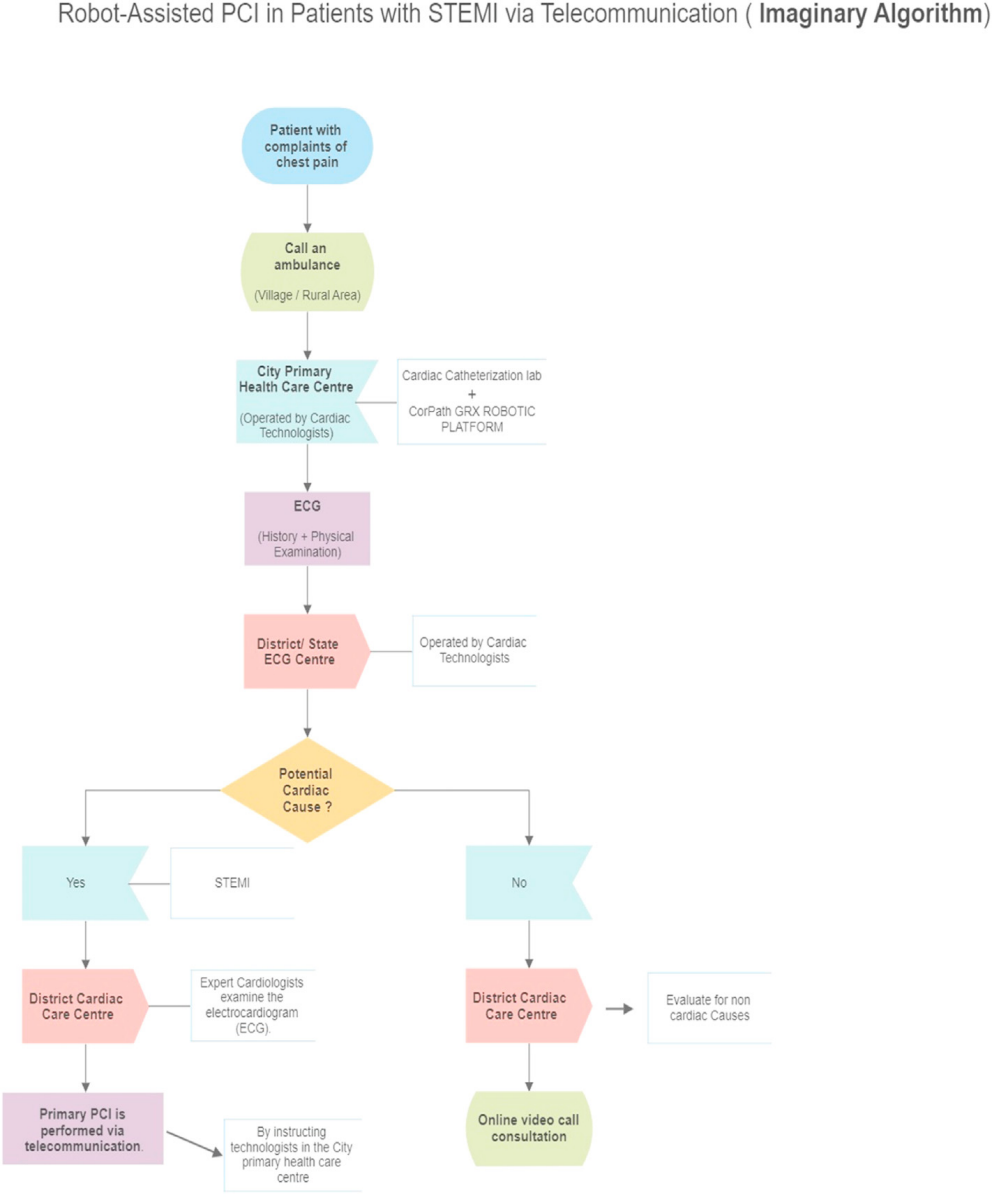
Imaginary algorithm For STEMI patients.

In many emerging economies, healthcare delivery varies greatly from major metropolitan areas to urban centres, semi-urban townships, and rural villages. Primary PCI is easily accessible in urban and suburban locations (although the cost of the treatment and road congestion constrains it). However, suburban town hospitals may have PCI accessibility challenges. Telestenting is an emerging technique in interventional cardiology.^28^ Remote tele-R-PCI through the telerobotic platform for STEMI patients in rural areas and during pandemic scenarios such as infectious disease transmission(Covid-19) may be viable. A more comprehensive explanation of the imaginary algorithm and its various steps is included in the following (Fig. 1).

1. If a patient presents with chest discomfort in a rural or village setting. “Call an Ambulance” is the first step in an imaginary algorithm. It is recommended that ambulance numbers be assigned separately for coronary care.
2. Establishment of a city primary health care facility with a well-equipped ECG and a cardiac catheterization lab operated by cardiac technologists. When the patient arrives at the city primary health care centre, he or she will be subjected to a physical examination and an ECG.
3. The ECG report will be transferred to the district ECG centre for approval (comprised of well-equipped remote ECG interference operated by a group of senior cardiac technologists).
4. A group of senior cardiac technologists from the district ECG centre will investigate the possible cause and notify or alert the District Cardiac Care Center (comprised of a well-equipped computer network -LAN or WAN operated by a group of senior cardiologists).
5. A group of senior cardiologists will review the reports submitted by the ECG centre in the District Cardiac Care centre. The primary PCI will be performed in the city's primary health care centre using a robotic platform with the guidance of senior cardiologists from the remote District Cardiac Care centre. Anticoagulation and antiplatelet therapy were administered per standard protocol.
6. In the case of patients with non-cardiac causes, a group of senior cardiologists in the District Cardiac Care centre will review the reports submitted by the ECG centre. The consultation will be conducted via online video call by senior cardiologists in the remote District Cardiac Care centre.

#### 6.6.1 Arterial access and diagnostic angiograms

**Fig. 2:**
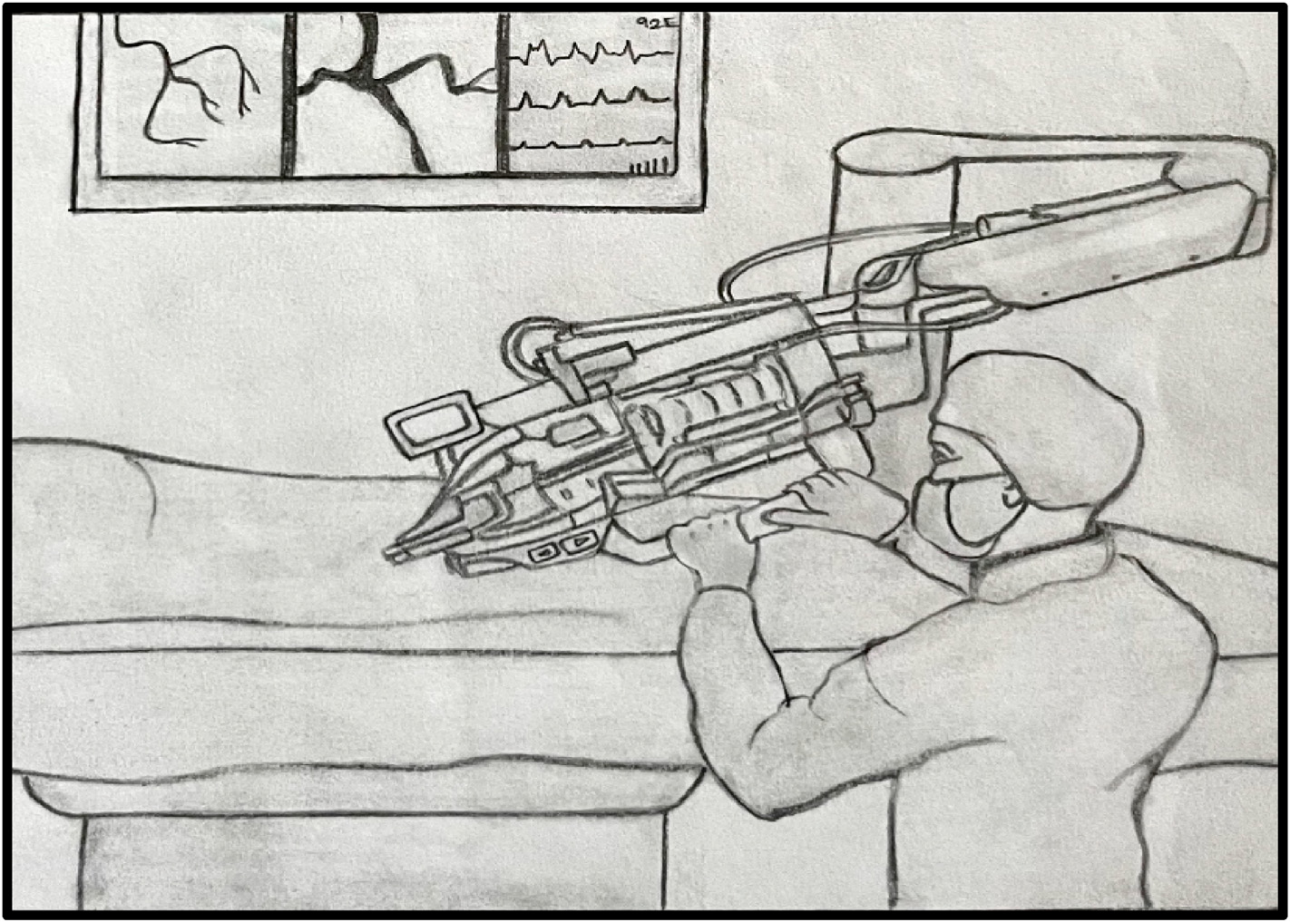
Primary health care center (bedside technologists).

A commercial endovascular simulator system may attempt PCI (Angio Mentor, Simbionix, Littleton, CO).^25^ The system simulates real-time PCI procedures and includes an access site for inserting interventional devices such as guide catheters, coronary guidewires, and balloon catheters. Following insertion, sensors in the simulator detect subsequent manipulations of these devices, such as advancement, retraction, and torque, and display them on the bedside fluoroscopy screen as corresponding movements of the virtual devices in the endovasculature of the simulated patient. The PCI procedure was performed using the robotic system “CorPath GRX, Corindus, Siemens Healthineers Company, Waltham, MA” (Fig. 5). The interventional cardiologist and robotic control system were located in a district cardiac care center. The PCI simulator and robotic drive were located in a primary health care centre, away from the interventional cardiologist in a district cardiac care centre. A cardiac technologist at the primary health care centre manually advanced a 6-French guide catheter into the ascending aorta, place a 0.014-inch coronary guidewire at the tip of the guide catheter, and loaded the guidewire and coronary balloon catheter onto the bedside robotic drive (Fig. 2). Further manipulation of the guide catheter, guidewire, and balloon catheter was performed remotely and robotically by an offsite interventional cardiologist at a district cardiac care centre. Interventional cardiologists can use remote-control systems to perform coronary angiography. This involves combining commercial endovascular simulation systems with robot-assisted coronary catheter manipulation.

#### 6.6.2 Infrastructure for RPCI

**Fig. 3:**
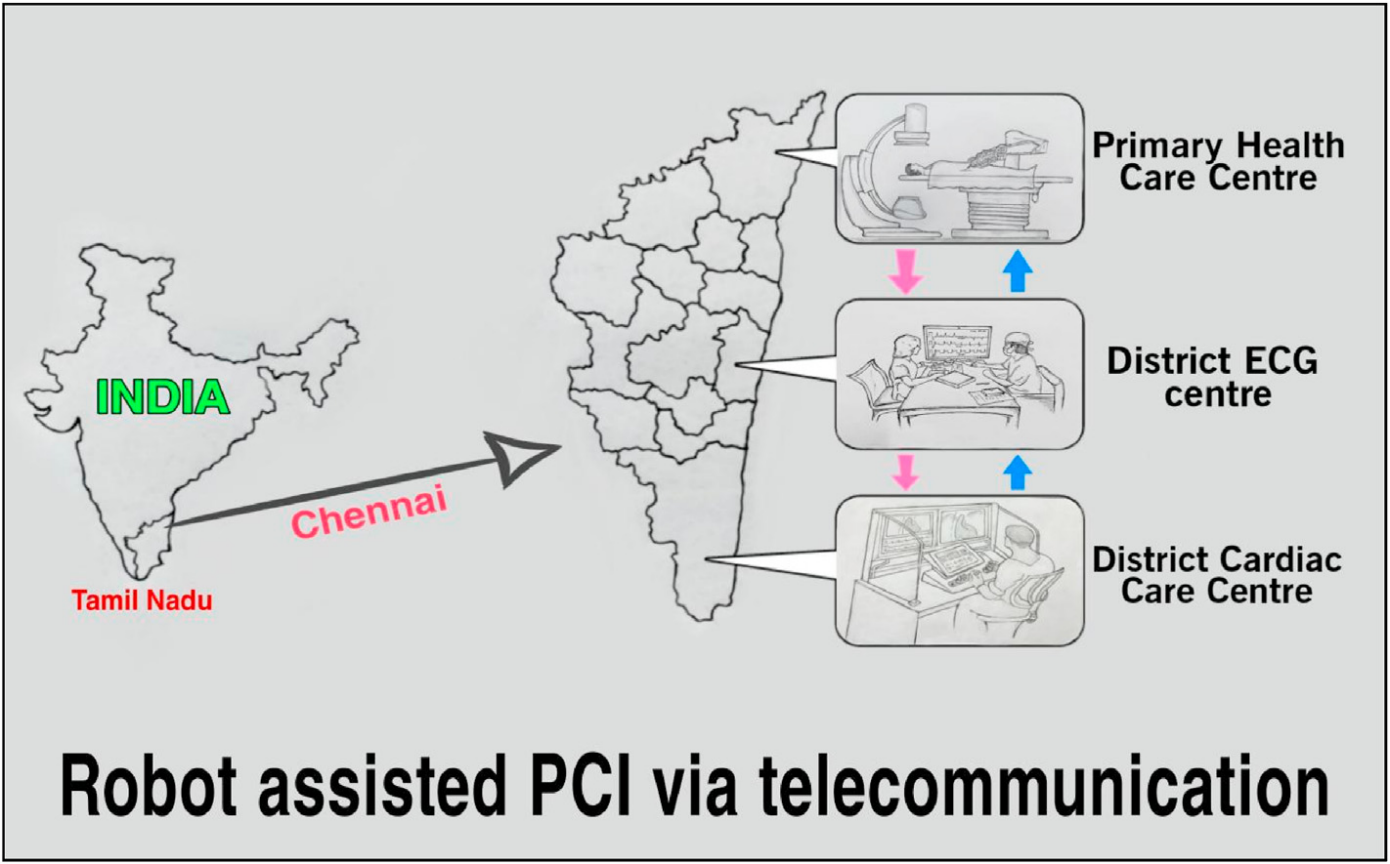
R–PCI via telecommunication.

**Fig. 4:**
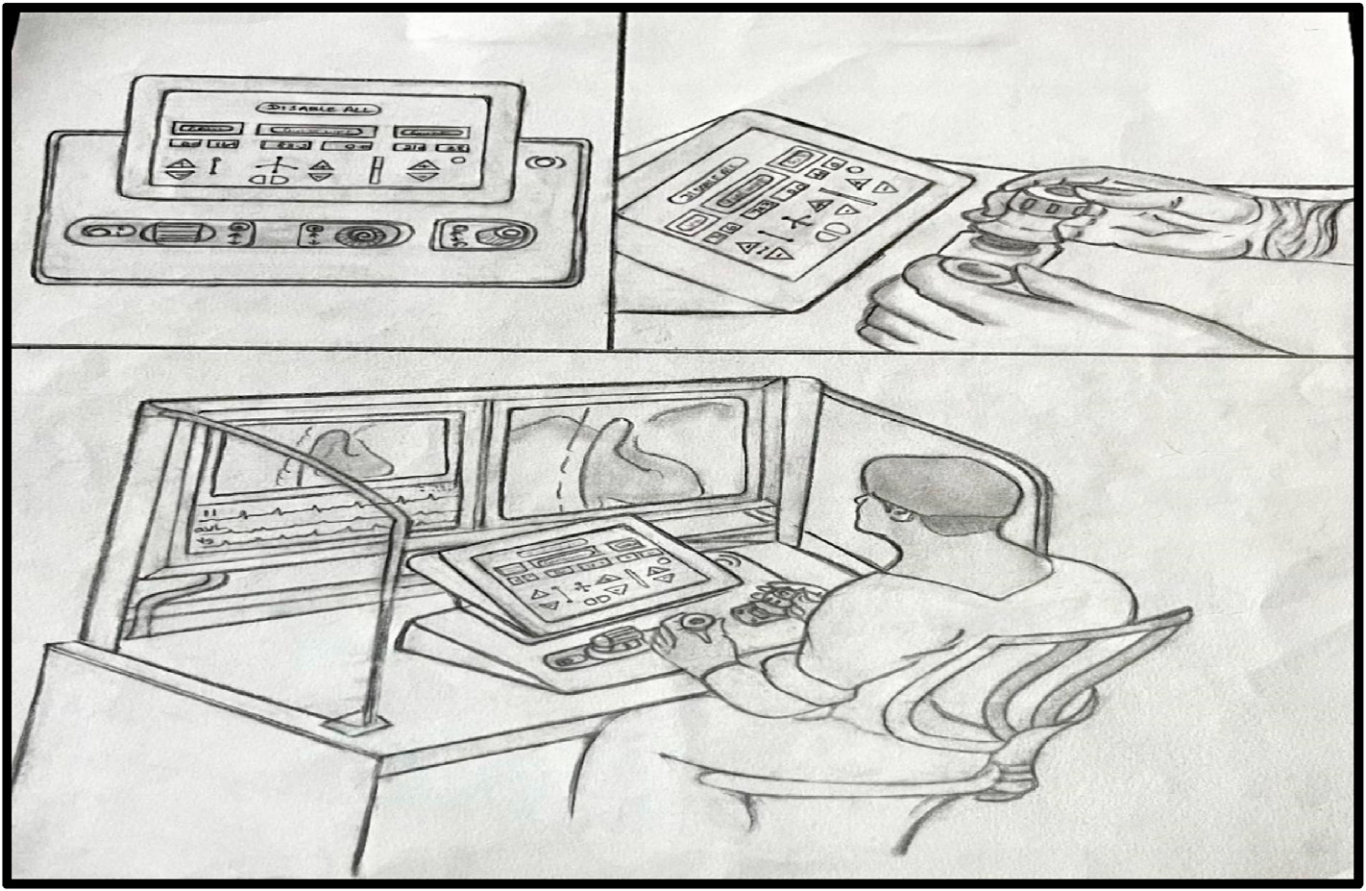
District cardiac care centre console.

The National Health Mission (NHM) is a government programme that funds the creation of primary health centres (PHCs) in rural and hilly areas, as well as tribal and desert areas. As of March 31, 2019, there were 1422 PHCs in rural areas and 463 PHCs in urban areas in Tamil nadu, India.^29^ The district has to set up two district cardiac care centres, one in the rural area and another in the urban area. Each district in Tamil Nadu will have to set up 20 primary healthcare centres with state-of-the-art equipment, including electrocardiographs (ECGs) and laboratories with remote robotic platforms. These centres will be in both urban and rural areas. Primary health centres in rural areas will receive instructions from the rural-district cardiac care centre, while those in urban areas will receive instructions from the urban-district cardiac care centre. The interventional cardiologists in rural and urban district cardiac care centres use a robotic system to manipulate coronary guidewires and balloon catheters in the rural and urban primary health care centres in a manner necessary to perform PCI on a simulator (Fig. 3, Fig. 4).

**Fig. 5:**
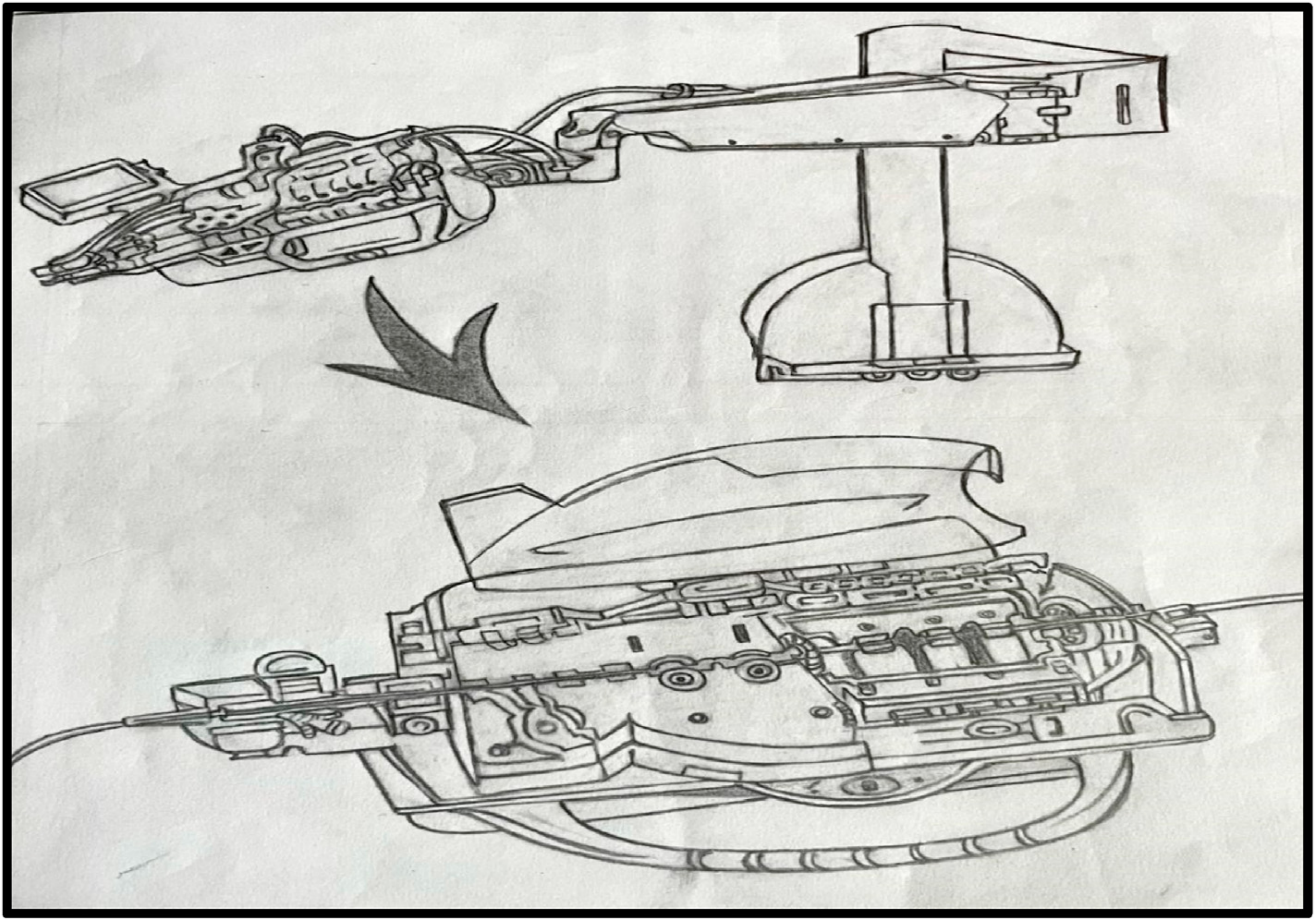
Robotic cassette.

#### 6.6.3 Telementoring: remote training

Telementoring is a form of training already being used in other areas of medicine, and it has the potential to help medical centres in underserved areas where access to education in interventional cardiology is limited. Mentors (experienced with robotic platforms) worldwide can remotely educate, train, and supervise interventional cardiologists using video conference capabilities. The live robotic PCI case demonstrations at interventional cardiology conferences can help the entire interventional community learn more about the procedures.

#### 6.6.4 Financial burden

Physicians are a significant cost factor in healthcare, especially in interventional cardiology.^30^ Major PCI-capable hospitals in remote areas require a 24-h, experienced interventionist. Each centre should have at least three or four intervention specialists on a shift basis. Robotic-assisted PCI requires highly skilled clinicians in limited numbers to perform the procedure remotely. The robotic system could help reduce the cost of cardiac catheterization labs, making PCI more affordable for patients.

### 6.7 Limitations

Limitations of R–PCI include current incompatibility with certain intravascular imaging catheters, FFR, thrombosuction, rotational atherectomy devices, embolic protection devices, etc. The inability to manipulate multiple guidewires and stents simultaneously, such as in treating bifurcation lesions or trifurcation lesions, is an important limitation of R–PCI. However, given these caveats, R–PCI remains a novel technology and has yet to become commonplace in cardiac cath laboratories. With increasing safety and feasibility data emerging, it is possible that R–PCI may form part of standard practice in the future.

## 7 Conclusion

In terms of procedure efficiency, operator radiation reduction, and safety, the recent implementation and development of second-generation robotic systems have significantly impacted interventional cardiology. This technology will play a significant role in the future of interventional cardiology as advancements eliminate the need for manual assistance and improve device compatibility. Additionally, advances in robotics will expand the use of tele-stenting procedures. A more extensive study demonstrating the safety and feasibility of tele-stenting over greater geographic distances and addressing fundamental technical difficulties would be required before attempting R–PCI.

## Funding

This study was carried out with no external sources of funding.

## Declaration of competing interest

All authors have no confict of interest to disclose.
